# Hybrid Stabilization of Thoracic Spine Fractures with Sublaminar Bands and Transpedicular Screws: Description of a Surgical Alternative and Review of the Literature

**DOI:** 10.1155/2015/857607

**Published:** 2015-11-16

**Authors:** Marie-Therese Unterweger, Frank Kandziora, Klaus J. Schnake

**Affiliations:** Center of Spinal Therapy, Schön Klinik Nürnberg Fürth, 90763 Fürth, Germany

## Abstract

Stabilization of unstable thoracic fractures with transpedicular screws is widely accepted. However, placement of transpedicular screws can cause complications, particularly in the thoracic spine with physiologically small pedicles. Hybrid stabilization, a combination of sublaminar bands and pedicle screws, might reduce the rate of misplaced screws and can be helpful in special anatomic circumstances, such as preexisting scoliosis and osteoporosis. We report about two patients suffering from unstable thoracic fractures, of T5 in one case and T3, T4, and T5 in the other case, with preexisting scoliosis and extremely small pedicles. Additionally, one patient had osteoporosis. Patients received hybrid stabilization with pedicle screws adjacent to the fractured vertebral bodies and sublaminar bands at the level above and below the pedicle screws. No complications occurred. Follow-up was 12 months with clinically uneventful postoperative courses. No signs of implant failure or loss of reduction could be detected. In patients with very small thoracic pedicles, scoliosis, and/or osteoporosis, hybrid stabilization with sublaminar bands and pedicle screws can be a viable alternative to long pedicle screw constructs.

## 1. Introduction

Indications for surgical interventions in thoracic fractures are neurological symptoms: feared neurological aggravation, unstable fractures, or unbearable pain with ongoing immobilization, respectively [1, 2].

Fractures of thoracic vertebrae are usually stabilized by an internal fixateur with transpedicular screws and rods. To achieve adequate biomechanical stability, long posterior constructs are recommended [2, 3]. Our treatment protocol includes stabilization of two vertebrae above the fractured one and two below with eight screws in total [1]. The screws are linked by two vertical rods and, if necessary, one cross link.

The anatomic specifics of the thoracic vertebrae often lead to problems in placing the pedicle screws. The pedicles are smaller [4] and formed differently [2]. Misplaced screws can lead to severe complications [5, 6].

The Universal Clamp System (Zimmer, Warsaw, USA) was developed as an advancement of the Luque Wiring [7] where sublaminar wires were placed around the lamina. The sharp wires could easily cause injuries of the spinal cord resulting in neurological deficiencies [8–10]. The Universal Clamp (UC) System is based on the same idea but uses flexible bands made from polyethylene. After the positioning of the band, it is fixed in a clamp which is fastened to the vertical rod. The system is typically used in deformity surgery and has been occasionally used in fractures [11, 12].

In comparison to multisegmental transpedicular stabilization, a so-called “hybrid stabilization” with sublaminar clamps and only four transpedicular monoaxial pedicle screws has the advantage that less screws have to be placed in the thoracic spine. With the hybrid stabilization the two vertebrae adjacent to the fractured one, the one above and the one below, are treated with pedicle screws and the two vertebrae next to them, again one cranial and one caudal, are fixed by sublaminar clamps.

We report for the first time two cases of thoracic fractures treated with the above described hybrid stabilization.

## 2. Patients and Methods

### 2.1. Surgical Technique

The sublaminar bands were used in combination with monoaxial (Medtronic, Minneapolis, USA) or polyaxial (Stryker, Kalamazoo, USA) pedicle screws of 5.5 mm diameter.

The vertebrae next to the fractured one were supplied with monoaxial or polyaxial transpedicular screws whereas the vertebrae next to these were fixed with sublaminar clamps.

First, the four necessary pedicle screws are set in a standard open technique under image intensifier control.

Then the vertebrae are prepared to pass the sublaminar bands. Therefore, the ligamentum flavum was partially removed. An arcuated dissector can help in checking the free space between the lamina and the dura. If the space is adequate, the sublaminar bands can be passed. First, the stiff part of the band is run in the clamp and then moved under the lamina from caudal to cranial. The band should always stay in contact to the lamina so the dura is not endangered. The surgeon has to verify that the band is flat against the lamina and that it is not twisted. Then the band is preassembled with the clamp and is pushed into the rod. The rods have to be prepared and should be long enough so they can hold the clamps and the screws. Anatomical bending of the rods has to be done before fixing them to the implants. After preparing the rods, the clamps can be connected to them. The rods should now connect the screws and all the clamps. The pedicle screws are fixed to the rods before fixing the sublaminar bands. With this maneuver the first step of reduction of the fracture can be ensured. The final reduction is achieved by using the reposition tool. The bands are strained until the clamps are properly fixed to the laminae. When all implants are set in the desired position, the clamp screws of the UC System are fixed and the bands are shortened.

## 3. Case Reports

### 3.1. Case 1

**Figure 1:**
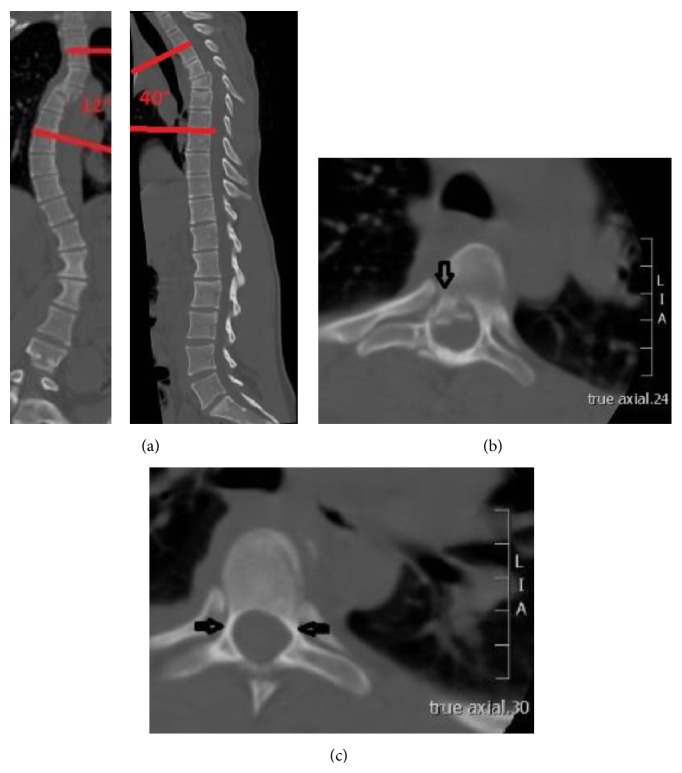
(a) CT scans showing the preexisting scoliosis and the fracture of T5. Cobb angle (T3–7): 12°, kyphosis angle (T3–7): 40°. (b) CT scan showing the fragment narrowing the spinal canal. (c) CT scan showing the small pedicles (blind on the right side, 2.5 mm on the left).

An 18-year-old woman fell from a horse and sustained an unstable rotation-flexion-burst fracture of the 5th thoracic vertebra (C 2.2 according to AO-Magerl classification [13]; Figure 1(a)) with narrowing of the spinal canal (Figure 1(b)). The patient had a preexisting idiopathic adolescent scoliosis.

She did not suffer from any neurological deficit.

Because of the instability and severe pain we decided to treat the patient surgically. We stabilized the thoracic spine from 3rd to 7th thoracic vertebra from posterior with pedicle screws and the sublaminar bands in the above described hybrid technique.

Since the patient had very small thoracic pedicles (Figure 1(c); 2.5 mm on the left side of the vertebra above the fractured one; on the right side the pedicles were blind, 1.6 mm in the vertebra below the fractured one), the only way to put the pedicle screws was parapedicular. The sublaminar bands were placed at the 3rd and 7th thoracic vertebra. Two rods were placed and fixed at the screws and the sublaminar clamps. A posterior fusion was added by using local bone graft together with demineralized bone matrix (DBM Pasty, Synthes, West Chester, USA) and tricalcium phosphate (chronOS, Synthes, West Chester, USA). No intraoperative complication occurred. The blood loss was 500 mL and the total operating time was 180 minutes.

The postoperative course was uneventful. The patient could be mobilized without orthosis. She could be discharged on the 10th postoperative day.

**Figure 2:**
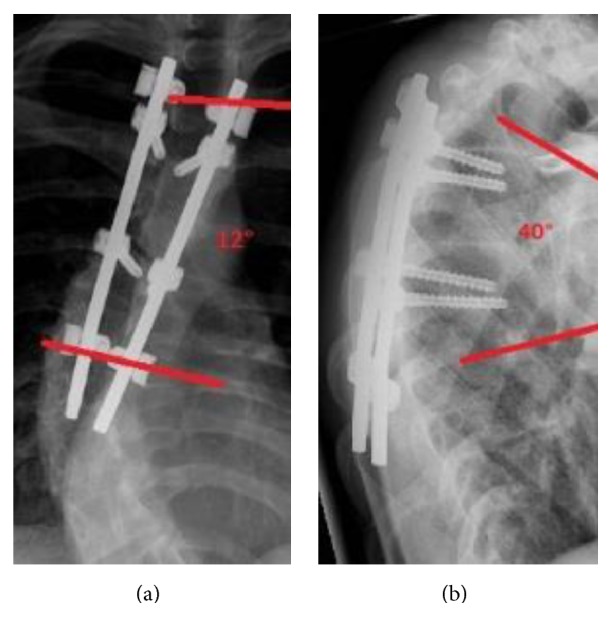
(a) Postoperative result in ap view with pedicle screws in T4 and T6 and UC System in T3 and T7 (12 months). Cobb angle (T3–7): 12°. (b) Postoperative result in lateral view (12 months). Kyphosis angle (T3–7): 40°.

At final follow-up after 12 months (Figures 2(a) and 2(b)) patients did not suffer from any back pain.

### 3.2. Case 2

**Figure 3:**
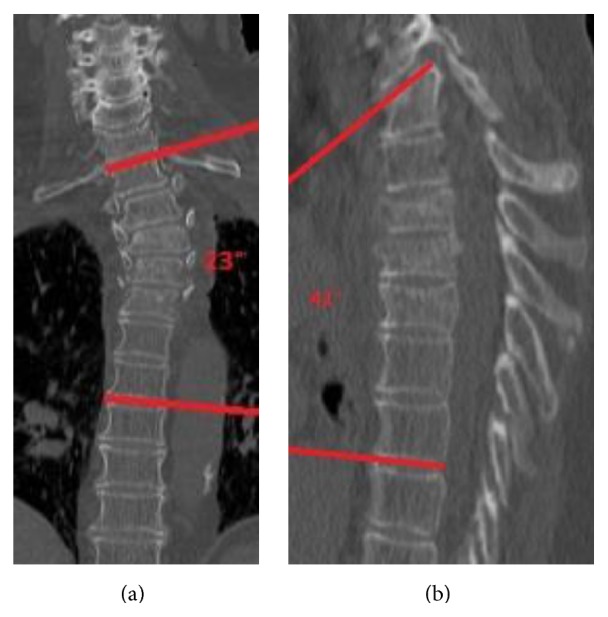
(a) CT scan showing the scoliosis and osteoporosis of the patient and the fractures in T3–T5. Cobb angle (T1–7): 23°. (b) CT scan in the sagittal profile. Kyphosis angle (T1–7): 41°.

A 75-year-old woman fell in a bus and sustained a multisegmental injury with fractures of the 3rd, 4th, and 5th thoracic vertebra (Figures 3(a) and 3(b)). According to AO-Magerl classification [13], the fractures were classified as A3.1, B2.3, and A3.1, respectively. She also had an existing cervical-thoracic right-convex scoliosis and an osteoporosis.

The patient did not have any neurological deficit. The injury was treated surgically because of the instability and the deformation. Reduction and stabilization were performed from the 1st to the 7th thoracic vertebra. The surgery was carried out one day after the accident. The 1st and 7th thoracic vertebrae were supplied by sublaminar bands whereas the 2nd and 6th thoracic vertebrae were supplied by polyaxial pedicle screws. A posterior fusion from T1 to T7 was initiated by demineralized bone matrix (DBM Pasty) and tricalcium phosphate (chronOS). No complication occurred. The blood loss was 550 mL and the total operation time was 280 minutes.

The postoperative course did not show any complicating events. The patient was mobilized without orthosis. She could be discharged to a geriatric rehabilitation clinic on the 12th postoperative day. When leaving the clinic she could walk with a walking frame by herself.

**Figure 4:**
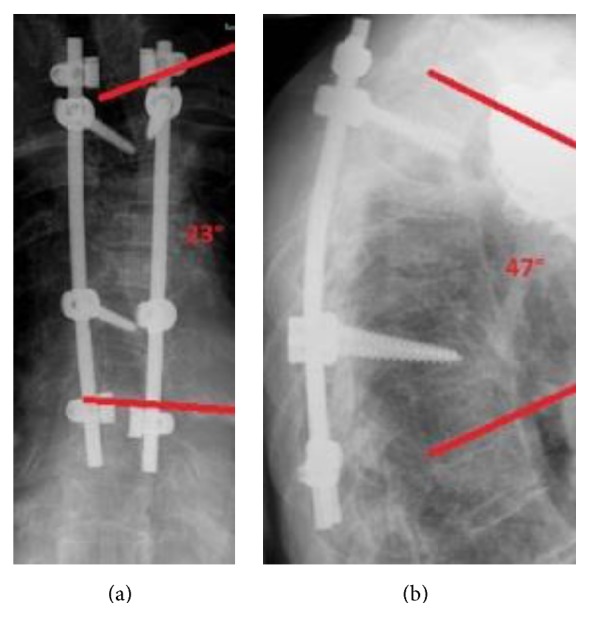
(a) Postoperative result with pedicle screws in T2 and T6 and UC System in T1 and T7 (12 months). Cobb angle (T1–7): 23°. (b) Postoperative result lateral (12 months). Kyphosis angle (T1–7): 47°.

At final follow-up at 12 months (Figures 4(a) and 4(b)) patient was ambulatory and complained about moderate back pain (VAS 4).

## 4. Discussion

The two cases illustrate the feasibility of the hybrid technique in thoracic fractures with difficult anatomical conditions. To the best of our knowledge, the used hybrid technique has not been published yet.

Gazzeri et al. [11] conducted a study with a different hybrid construct and stabilized thoracolumbar vertebrae with pedicle screws and UC System. Some patients suffered from a vertebral fracture, and the surgeons implanted screws in two to three segments underneath the fracture and sublaminar bands above the fractured vertebra. Our construct differs in the formation of the sublaminar bands and pedicle screws. The biomechanical characteristics of the implants are well distributed, so the stronger pedicle screws with a biomechanical failure strength of 1000 N [14] are adjacent to the fracture, potentially resulting in less loss of reduction.

Long constructs are widely recommended in thoracic fractures and have advantages over the short-segment stabilization concerning the biomechanical stability and loss of correction [1, 2]. McLain [3] claimed that long-segment stabilization has different advantages when used in thoracic fractures. Beside others, the advantages of the long constructs are the multiple fixation points which distribute the forces necessary for the correction over a greater number of segments. So, the force on every point is reduced and the risk of pullout failure is minimized [3]. Disch et al. [15] studied the biomechanical stability of different types of stabilization after spondylectomy and showed that, in the thoracolumbar spine, the long-segment stabilization has a higher stiffness in all motion planes.

Placing thoracic screws in the thoracic vertebrae often is difficult because of the special anatomic features of the thoracic pedicles. The pedicles have smaller diameters compared to the rest of the thoracolumbar spine [16]. Typically, the smallest ones can be found in the 4th thoracic vertebra with an average of 4.5 ± 1.2 mm [17]. If the screw has a bigger diameter than 80% of the pedicle, it can cause morphological changes of the pedicles [18]. This can result in pedicle fractures, breakout of the screws, and extension of the pedicle.

A burst fracture of the pedicle simplifies the screw breakout [14]. In weak bone especially, like in patients suffering from osteoporosis, the risk of a breakout is higher than that in healthy bone [5, 19].

The consequences of the above-mentioned anatomical situation are frequently misplaced pedicle screws, mostly in the lateral direction because of the thinner pedicle wall on the lateral side [6, 20].

In the literature the frequency of misplaced thoracic screws is reported to be up to 40% [3, 21–23]. Misplaced screws can injure the dura or the spinal cord. They can also impinge the nerve root with associated neurological deficiencies. Other complications are the injury of organs, vessels, or nerves [5, 6, 24–28].

With the proposed hybrid stabilization, the risk of screw misplacement is lowered due to the simple fact that less screws have to be placed.

In special cases, the anatomy of the thoracic vertebrae can be even more challenging, for instance in deformed spines like scoliosis, osteoporosis, and deformation of the vertebrae, like in wedge-shaped vertebrae after a former fracture.

Patients who are suffering from a scoliosis have small pedicles on the concave side of the spine, especially on the apex and the main curve. Therefore, some authors suggest that the screw insertion on the apex of the curve should be avoided [14]. Pedicle diameters are declared between 2.5 and 4.0 mm [29–32]. Kotani et al. [33] reported a pedicle perforation in 11% of the observed patients with a scoliosis.

In our case the patient had a preexisting scoliosis with pedicle diameter less than 3 mm. Placing the sublaminar bands was therefore much easier and safer than inserting the pedicle screws.

Hybrid stabilization with pedicle screws and sublaminar bands might be beneficial in patients with osteoporosis. Chao et al. [19] showed in a biomechanical study that in patients with a *T*-score of −5.2 the pull-out force amounts to 144.3 ± 92.1 N. In comparison, in healthy bone, the pull-out force accounts for around 1000 N [5]. In osteoporotic spines, the posterior part with the lamina is stronger than the anterior parts [34]. So, the lamina should be an ideal part to fix an implant. The Universal Clamp System showed high failure loads of 401 ± 120 N in fresh frozen human thoracic spines [35]. In our case number two, no postoperative implant failure occurred despite severe osteoporosis and long construct. No cement augmentation of the screws was necessary.

Another problem for the surgeon is the fact that it can be difficult to visualize bony thoracic structures with the image intensifier intraoperatively. When using sublaminar bands, no radiological control is necessary.

Also the use of sublaminar bands has some limitations. First, a decompression is necessary which carries the risk of dural tears or even damaging the cord. If a laminectomy is performed, no clamps can be fixed at this level. Finally, inserting the sublaminar band takes more time than placing a pedicle screw, at least in our hands. A slightly prolonged surgical time should be considered.

Limitations are the short follow-up time of one year and the missing CT scan after one year. So the fusion rate cannot be determined for sure.

## 5. Conclusion

In patients with a combination of an unstable fracture and difficult anatomic conditions the hybrid stabilization with sublaminar bands and pedicle screws is a reliable technique. Scoliosis, osteoporosis, or small pedicles are risk factors for pedicle screw failure. The use of sublaminar bands can help to avoid such complications.
